# Inhibitory Effects of Ethyl Gallate on *Streptococcus mutans* Biofilm Formation by Optical Profilometry and Gene Expression Analysis

**DOI:** 10.3390/molecules24030529

**Published:** 2019-02-01

**Authors:** Vika Gabe, Tomas Kacergius, Saleh Abu-Lafi, Povilas Kalesinskas, Mahmud Masalha, Mizied Falah, Basheer Abu-Farich, Andrius Melninkaitis, Mouhammad Zeidan, Anwar Rayan

**Affiliations:** 1 Department of Physiology, Biochemistry, Microbiology and Laboratory Medicine, Institute of Biomedical Sciences, Faculty of Medicine, Vilnius University, 03101 Vilnius, Lithuania; vika.gabe@mf.vu.lt (V.G.); tomas.kacergius@mf.vu.lt (T.K.); povilas.kalesinskas@mf.vu.lt (P.K.); 2 Faculty of Pharmacy, Al-Quds University, Abu-Dies 144, Palestine; sabulafi@staff.alquds.edu; 3 Microbiology Laboratory, QRC-Qasemi Research Center, Al-Qasemi Academic College, P.O. Box 124, Baka EL-Garbiah 30100, Israel; mahmudmasalha@gmail.com; 4 Galilee Medical Center, P.O. Box 21, Nahariya 22100, Israel; MiziedF@gmc.gov.il; 5 Faculty of Medicine in the Galilee, Bar-Ilan University, Ramat Gan 5290002, Israel; 6 Science department, Al-Qasemi Academic College, P.O. Box 124, Baka EL-Garbiah 30100, Israel; af_basheer@qsm.ac.il; 7 Laser Research Center, Vilnius University, Vilnius 10223, Lithuania; andrius.melninkaitis@ff.vu.lt; 8 Molecular Genetics and Virology Laboratory, QRC-Qasemi Research Center, Al-Qasemi Academic College, P.O. Box 124, Baka EL-Garbiah 30100, Israel; mouhammad.zeidan7@gmail.com; 9 The Institute of Applied Research—Galilee Society, P.O. Box 437, Shefa-Amr 20200, Israel; 10 Drug Discovery Informatics Lab, QRC—Qasemi Research Center, Al-Qasemi Academic College, Baka El-Garbiah 30100, Israel

**Keywords:** ethyl gallate, *Streptococcus mutans*, biofilm, acidogenicity, gene expression, natural product

## Abstract

This study aimed to test the effectiveness of ethyl gallate (EG) against *S. mutans* biofilm formation on solid surfaces (polystyrene, glass) and acidogenicity, and to examine the effect on expression of related genes. The biofilm that is formed by *S. mutans* bacteria was evaluated using colorimetric assay and optical profilometry, while the pH of the biofilm growth medium was measured with microelectrode. The expression of genes encoding glucan binding protein B (*gbpB*), glucosyltranferases B, -C, -D (*gtfB, -C, -D*) and F-ATPase (*atpD*, *atpF*) was assessed using a quantitative reverse transcription-polymerase chain reaction (RT-qPCR). It was revealed that all of the EG concentrations significantly suppressed *S. mutans* biofilm build-up on polystyrene and glass surfaces, and inhibited acidogenicity, in a dose-dependent manner, compared to the activity of untreated bacteria (*p* < 0.05). The highest concentration of EG (3.53 mM) reduced biofilm formation on polystyrene and glass surfaces by 68% and more than 91%, respectively, and prevented a decrease in pH levels by 95%. The RT-qPCR data demonstrate that the biofilm-producing bacteria treated with EG underwent significant gene expression changes involving the *gtfC* (a 98.6 increase in fold change), *gtfB* gene (a 47.5 increase in fold change) and the *gbpB* gene (a 13.8 increase in fold change). However, for the other genes tested (*gtfD*, *atpD* and *atpF*), the EG treatments did not produce significant expression change compared to the control. EG produced significant gene expression change in three genes—*gtfC,* *gtfB*, and *gbpB*; it has the capacity to inhibit *S. mutans* biofilm formation on solid surfaces (polystyrene, glass), as well as acidogenicity. Therefore, EG might be used as an antibiofilm and/or anticaries agent for oral formulations in order to reduce the prevalence of dental caries.

## 1. Introduction

The production of biofilm, also known as dental plaque, is a virulent action of *Streptococcus mutans* on tooth surfaces [1,2] Five essential metabolic pathways are involved in cariogenic biofilm produced by *S. mutans*. These pathways are regulated by several known genes; they include (1) for microbial adhesion, *gbpB*, *sacB* (*ftf*), *vicR* and *wapA*, which are involved in sucrose-dependent adhesion, and *spaP*, involved in sucrose-independent adhesion [3,4,5]; (2) for biofilm formation, *atlA*, *sacB* (*ftf*), SMU.609, *vicR* and *wapA* [6,7,8]; (3) for extracellular polysaccharide synthesis, *gtfA*, *gtfB*, *gtfC*, *gtfD*, *sacB*, (*ftf*) and *vicR* [4,5]; (4) for carbohydrate uptake, *mipB*, SMU.104, SMU.105 and *sorA* [9,10]; and (5) for acid tolerance, comD and SMU.1037c [11]. Several groups of proteins and enzymes are encoded by these genes; these include: the glucosyltransferases (GTFs) GTFB and GTFC, which synthetize water-insoluble glucans with α-1,3-glucosidic linkages, and GTFD, which synthesizes water-soluble glucans rich in α-1,6-glucosidic linkages; glucan-binding proteins (Gbp proteins); the cell surface protein antigen (PAc); stress response proteins (RecA, DnaK and GroEL); collagen-binding proteins (cnm, cbm); the two component proteins of the quorum sensing system; and F-type ATPases, which are considered one of the most important components of the acid tolerance response (ATR) that gives *S. mutans* a significant competitive advantage over other species under acidic conditions. *S. mutans* can strongly adhere to teeth via synthesis of a glucan matrix and can rapidly dominate dental plaque by using glycolytic end-products to acidify the microenvironment and kill competitors [12,13]. The F_0_F_1_-H/F-ATPase β subunit of the F_1_ protein, which is encoded by the *atpD* gene of *S. mutans,* has a lower optimal pH than that of many other oral microbes and is significantly upregulated during growth under acidic conditions, contributing to the relative aciduricity of the organism [14,15,16]. Altogether, these bacterial protein associations facilitate dental plaque production and dental caries induction. In this study, we investigated the effects of ethyl gallate (EG) on the expression of six representative genes and report on changes in the expression of three important genes—*gtfB, gtfC* and *atpD—*which are essential for biofilm production and maintenance. It should be noted that the selected *gtfB, -C, -D* genes are important for sucrose-dependent *S. mutans* biofilm formation leading to dental plaque production and subsequent dental caries induction, whereas *atpD* and *atpF* genes are important for acidogenicity of the biofilm leading to tooth cavitation because organic acids produced in the dental biofilm within mouth are in direct contact with tooth enamel causing demineralization of the tooth hard tissues (enamel and dentin).

Infections caused by resistant bacteria are currently on the rise and, hence, are considered a genuine health threat [17,18]. Natural plants and dark fruits are rich in antibacterial phytochemicals, such as polyphenols, and researchers are becoming more interested in isolating their secondary metabolites to inhibit the growth of pathogenic bacteria, especially streptococci. More attention has been paid to identifying phytochemicals that can combat *S. mutans*, due to its association with oral carcinoma [19]. Polyphenolic-rich extracts of various freshly prepared teas, such as green tea, black tea, grape seed, sloe berry skin, blackberry, pomegranate skin, black currant and hawthorn berry skin were tested for their activity against oral *streptococci* (various strains of *S. mutans*). Among the teas tested, red grape seed-extract displayed the most potent action against *S. mutans* [19]. Polyphenolic gallates are secondary metabolites derived from plants and are made up of esters of gallic acid. At present, EG is gaining much attention as a promising antioxidant compound [20]. In grape-seed-extract, gallic acid and EG were the two compounds that exhibited the strongest antimicrobial activity [21,22]. EG has been identified as an active component of *Pistacia integerrima Linn* [23]. This work aimed to investigate the effects of the bioactive phytochemical EG on biofilm formation by using an optical profilometry assay and to identify the genes associated with this bioactivity.

## 2. Results and Discussion

Recent studies have investigated the effects of pure phenolic compounds and extracts on the growth of pathogenic bacteria; these compounds have mainly been derived from grape seed, which is considered a rich source of polyphenolic compounds [21,24] Gallic acid and ethyl gallate are the compounds that have shown the greatest antimicrobial activity, which may be attributed to the three hydroxyl groups at the phenyl ring (pyrogallol group) [21]. A more recent study utilizing high-resolution LCMS, along with fluorescence detection, to determine the phenolic composition of 17 monocultivar, commercial, cold-pressed grape seed-oils, revealed the presence of ethyl gallate in nine of them, at a low concentration of 0.59 ppm [24]. This signifies the importance of the extraction process and the amount of seed that is used to satisfactorily quantifying important polyphenolic compounds.

**Figure 1:**
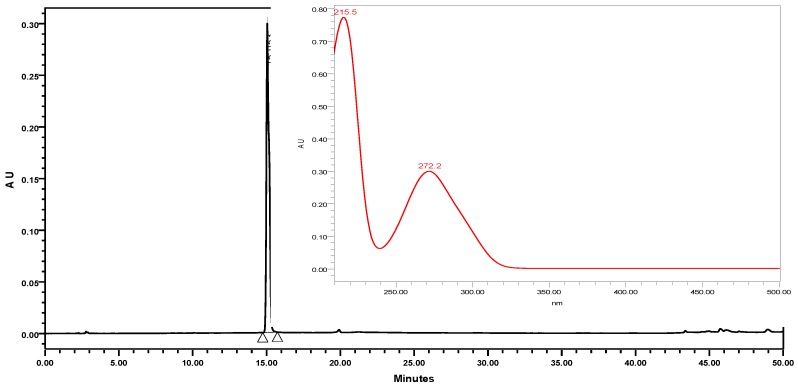
Chromatogram of standard EG, its corresponding structure, and ultraviolet-visible spectrum (210–500 nm).

A typical HPLC-PDA chromatogram of EG, its chemical structure, and its corresponding UV-Vis spectrum in the range of 210–500 nm using HPLC-PDA are shown in Figure 1. 

Five freshly prepared extracts derived from grape seed (containing water, methanol, ethanol, ethyl acetate and hexane), were injected into the HPLC under the same experimental conditions. The retention time and spectrum of standard EG were used to detect the presence of EG in the sample plants. At the same retention levels, UV-Vis spectral matching revealed very small concentrations of EG in the selected plants, in agreement with the findings of a recent study by Cecchi et al. [24]. Moreover, the chromatograms showed many gallates that possessed typical absorption maximums at approximately 272 nm. 

### 2.1. Antibacterial Activity of Ethyl Gallate (EG) on S. mutans and Determination of the MIC and MBC Values 

As shown in Table 1, the values of the MIC and MBC for the effects of EG on *S. mutans* are 1.56 mg/mL (7.87 mM) and 6.25 mg/mL (31.54 mM), respectively. The ratio between the two values is 1:4. 

### 2.2. Effects of EG on S. Mutans Biofilm Formation on Polystyrene Surfaces

**Figure 2:**
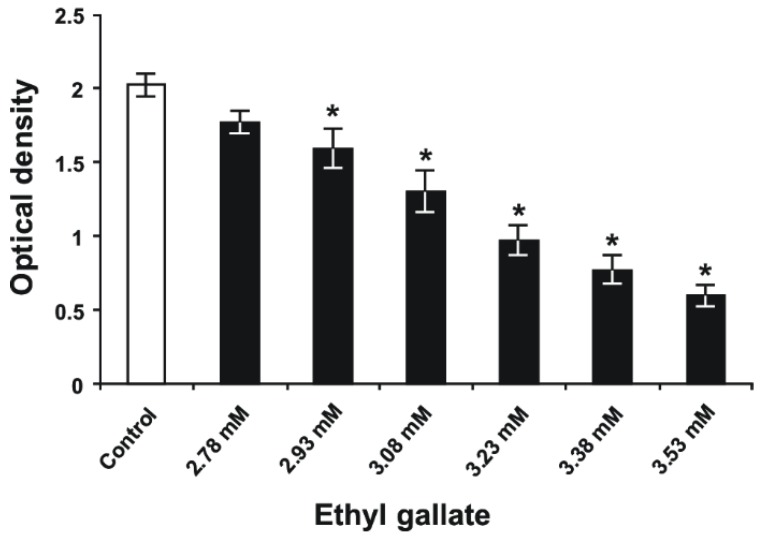
*S. mutans* biofilm biomass formed on polystyrene surfaces after 24 h of incubation in THB containing 1% sucrose and several different concentrations of EG. (Data are presented as the mean ± standard error for three independent experiments [*n* = 3−9]). *p < 0.05 when compared to the control group.

The MIC of EG was 1.56 mg/mL (7.87 mM) and based on that, the concentrations of EG, which were used for the biofilm inhibition, were lower than the determined MIC. The main point was to find the concentrations of EG that inhibit the biofilm formation but do not inhibit the growth of bacteria. Reasonably, this biofilm-inhibiting concentration should be lower than the determined MIC. As presented in Figure 2, EG concentrations of 2.93, 3.08, 3.23, 3.38, and 3.53 mM significantly reduced *S. mutans* biofilm biomass on polystyrene surfaces, unlike untreated bacteria (*p* < 0.05) in Todd Hewitt broth (THB) containing 1% sucrose. The antibiofilm activity of EG occurred in a dose-dependent manner, exhibiting its highest effect at a concentration of 3.53 mM, which decreased *S. mutans* biofilm formation by 68%, compared to the biofilm formation of untreated bacteria. However, the latter concentration of EG was unable to fully suppress *S. mutans* biofilm development on polystyrene surfaces. To date, there are no studies reporting the inhibitory effect of EG on the production of *S. mutans* biofilm biomass, except the investigation of Bakr et al. [25] that demonstrated such activity of EG isolated from *Nymphaea alba* L. rhizomes on the development of *Staphylococcus aureus* biofilm in vitro.

### 2.3. Effects of EG on S. mutans Biofilm Formation on Glass Surfaces 

**Figure 3:**
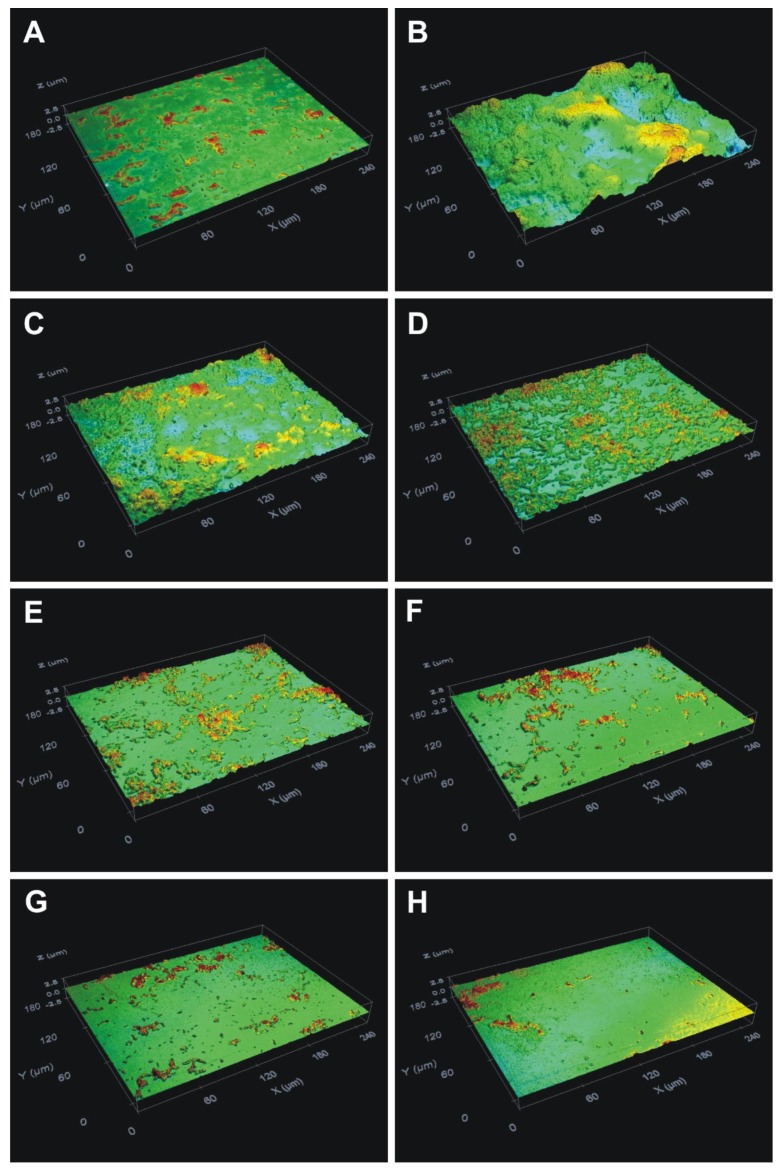
Optical profile of the glass slides with *S. mutans* culture biofilm after 24 h of incubation in the presence of different concentrations of ethyl gallate. Glass slide surfaces with bacteria incubated (**A**) without ethyl gallate, in the absence of sucrose and (**B**) without ethyl gallate, in the presence of 1% sucrose, and surfaces treated with (**C**) 2.78 mM, (**D**) 2.93 mM, (**E**) 3.08 mM, (**F**) 3.23 mM, (**G**) 3.38 mM, and (**H**) 3.53 mM of ethyl gallate. Magnification, ×50.

**Figure 4:**
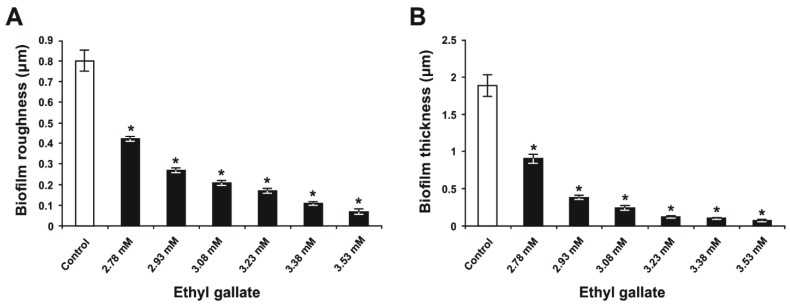
Quantities of *S. mutans* biofilm formed on the glass slide surfaces after 24 h of incubation in THB containing 1% sucrose and different concentrations of ethyl gallate. (**A**) the surface roughness parameter (*R*_q_) of the biofilm on the glass slides and (**B**) the biofilm thickness. Data are presented in terms of the mean ± standard error from three independent experiments (*n* = 18, biofilm roughness; *n* = 15, biofilm thickness). *p < 0.05 when compared to the control group.

The optical profilometry technique, applied in the analysis of the surfaces of the glass slides with *S. mutans* biofilm, was used to clarify and confirm the results of the colorimetric assay. First, unlike the condition of bacteria incubated without EG and sucrose (Figure 3A), where the *R*_q_ and thickness parameters for the untreated bacteria grown without sucrose were 0.07 ± 0.01 and 0.1 ± 0.01 μm, respectively, the presence of 1% sucrose in THB induced adherence of the bacteria to the glass slides and the subsequent maturation of biofilm (Figure 3B). Second, in the THB with 1% sucrose, exposure of *S. mutans* to 2.78, 2.93, 3.08, 3.23, 3.38, and 3.53 mM of EG (Figure 3C–H, respectively) decreased the formation of biofilm on the glass surfaces in a dose-dependent manner. Quantification revealed that the surface roughness parameter (*R*_q_) of the biofilm (Figure 4A) and the biofilm thickness (Figure 4B) were increased for the control bacteria (Figure 4); however, EG treatment inhibited this effect in a dose-dependent manner (*p* < 0.05; Figure 4). In this respect, EG concentrations of 3.23, 3.38, and 3.53 mM exhibited the greatest effects for reducing the surface roughness parameter (*R*_q_) of the biofilm, by 79%, 86%, and 91%, respectively (Figure 4A). Furthermore, these concentrations of EG decreased *S. mutans* biofilm thickness by 94%, 95%, and 96%, respectively (Figure 4B). Thus, the EG concentration at 3.53 mM almost completely inhibited *S. mutans* biofilm formation on the glass surfaces, in contrast to the biofilm formation on the polystyrene surfaces. Taking into consideration that EG is the ester of gallic acid, these findings are like the data obtained in the study performed by Kacergius et al. [26] that showed such *S. mutans* biofilm inhibiting effect by optical profilometry using another ester of gallic acid–methyl gallate (MG). However, it is important to note that in the present investigation lower concentration of EG was needed to completely suppress *S. mutans* biofilm formation on glass surface in comparison with the concentration of MG, i.e. 0.7 mg/mL (3.53 mM) versus 1 mg/mL. Hence, the EG is more effective for inhibition of *S. mutans* biofilm formation than the MG.

### 2.4. Effects of EG *on S. mutans* Biofilm Acidogenicity

The pH measurements of the *S. mutans* biofilm growth medium demonstrated that the bacteria grown in THB with 1% sucrose produced organic acids from the fermentation of this carbohydrate, leading to an ~1.8-fold decrease in pH, compared to the pH of the blank group (Table 2). This substantial decrease in pH indicates increased acidogenicity in the *S. mutans* biofilm. However, the treatment of *S. mutans* bacteria with EG significantly prevented a decrease in the pH level, as opposed to that of the untreated bacteria grown in THB with 1% sucrose (*p* < 0.05), by increasing pH close to the pH levels of the blank group (Table 2). This preventive effect of EG occurred in a dose-dependent manner, and EG concentrations from 3.38 to 3.53 mM increased the pH by 95–96%. Hence, the EG was able to almost completely inhibit the acidogenicity of *S. mutans* biofilm. These findings are similar to the data obtained in the study performed by Kacergius et al. [26] that showed such acidogenicity inhibiting effect of *S. mutans* biofilm using another ester of gallic acid–methyl gallate (MG). However, this effect occurred at lower concentrations of EG as compared to the MG concentrations.

### 2.5. Gene Expression

**Figure 5:**
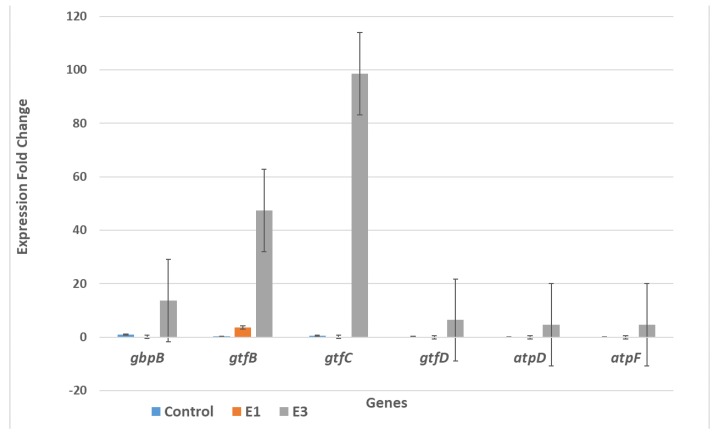
EG effects on the expression of six *S. mutans* genes that are involved in biofilm production. The *S. mutans* cells were collected from the biofilm phase. E1 means treatment with EG at a 1.56 mg/mL concentration (comparable to the MIC value), while E3 means treatment with EG at a 0.39 mg/mL concentration (comparable to 25% of the MIC value).

**Figure 6:**
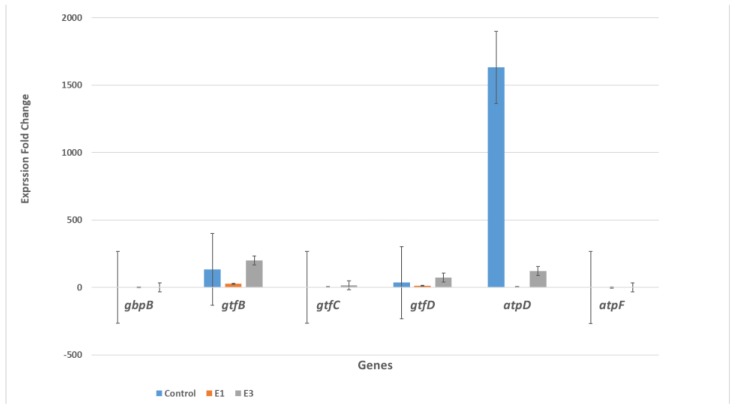
EG effects on the expression of six planktonic *S. mutans* genes that are involved in biofilm production. The *S. mutans* cells were collected from planktonic growth. E1 means treatment with EG at a 1.56 mg/mL concentration (comparable to the MIC value), while E3 means treatment with EG at a 0.39 mg/mL concentration (comparable to 25% of the MIC value).

SYBR qRT-PCR was used to determine the relative change in expression of six genes—*gbpB*, *gtfB*, *gtfC*, *gtfD*, *atpD* and *atpF*—after treating seeded *S. mutans* cells producing biofilm and planktonic cells with two concentrations of EG (1.56 mg/mL and 0.39 mg/mL) in comparison with control cells (untreated). Gene expression fold changes are shown in a bar graph in Figure 5 for the biofilm-producing cells and in Figure 6 for the planktonic cells. Figure 5 shows that the biofilm-producing cells treated with EG at a concentration of 0.39 mg/mL (labeled E3 and equal to 25% of the MIC value) exhibited significant gene expression changes in certain genes: *gtfC* (a 98.6 increase in fold change), *gtfB* gene (a 47.5 increase in fold change), and *gbpB* (a 13.8 increase in fold change). These results are in agreement with results of Deker et al. [27], who found that *gtfC* and *gtfB* were up-regulated in the presence of xylitol; however, our results show a higher factor of upregulation than was obtained with xylitol treatment. As for the other genes tested (*gtfD*, *atpD* and *atpF*), the EG treatments did not produce any significant expression change in comparison with the control. The effect of EG on the overexpression of *gtfC*, *gtfB* and *gbpB* could be attributed to stress induced by EG. To ameliorate this stress, bacteria respond with high gene expression for enzymes that synthesize water-insoluble glucans, which are essential for establishing a matrix that increases the coherence of bacterial cells and their adherence to surfaces and increases mechanical stability by binding bacterial cells together. In order for this to happen a concomitant increase in *gtfD*, *atpD* and *atpF* expression is needed. However, our results show that such a concomitant increase did not happen, and this might have disturbed the balance needed to increase biofilm production. Another possibility is that the increase in gene expression obtained upon EG treatment could be a result of disturbing the activity of regulators, quorum sensing component genes such as VicK, and products, as shown by Senadheera et al. [4], who witnessed the upregulation of *gbpB*, *gtfB*, and *gtfC* upon *vicRKX* overexpression. In contrast, Figure 6 shows that EG treatment of the planktonic cells caused very significant down-regulation for the expression of the *atpD* gene only (a 1631-fold change in the non-treated and a 121.7 fold change for the E3 treated). The *atpD* gene encodes for the β subunit of F_1_ protein F_0_F_1_-H/ATPase anchored to the cell membrane. Altogether, our results show that the biofilm and planktonic cells were affected differently by the EG treatment. However, the results show a discrepancy from the colorimetric, profilometric, and pH measurements. Another explanation for these results could be that the inhibition of biofilm formation on polystyrene and glass surfaces, and acidogenicity, by ethyl gallate occurs at the protein level and is not influenced at the level of gene expression. 

Therefore, to ascertain the direct effect of EG on the gene expression of biofilm-producing cells of *S. mutans*, more studies will be conducted in the future to follow the expression of larger number of genes known to be involved in biofilm production and maintenance, on the genetic level as well as on the protein level. 

## 3. Materials and Methods

### 3.1. The Source of Chemicals

Acetonitrile (ACN) HPLC-grade solvent was purchased from Merck (Darmstadt, Germany). Highly purified water was prepared with a Millipore Milli-Q Plus water purification system. Ethyl gallate, analytical standard, was purchased from Sigma, Rehovot, Israel.

### 3.2. Plant Extraction 

To perform the extraction, one gram of grape seed was packed in a tube, soaked with 15 mL of solvent (water, methanol, ethanol, ethyl acetate, and hexane), sonicated for 75 min at 40 °C, and then left in for 3 h to cool down. After complete extraction, the extract solution was filtered with Whatman paper, grade 1, and tested for its content of ethyl gallate by HPLC.

### 3.3. Instrumentation and Chromatographic Conditions

A Waters Alliance e2695 separations module, a 2998 photo diode array (PDA), and Empower 3 software was used (Waters, Eschborn, Germany). Ethyl gallate (EG) was run on a Waters HPLC ODS column (XBridge, 4.6 ID × 150 mm, 5 μm) with a guard column (Xbridge ODS, 20 mm × 4.6mm ID, 5 μm). The mobile phase consisted of water and acetonitrile (ACN) binary solvent mixture in the gradient mode, as follows: 95% water and 5% ACN at 0 min×, held for 2 min×, then raised to 50% water and 50% ACN over 15 min, then to 10% water and 90% ACN over 1 minute, held there for 3 min, and finally returned to 95% water and 5% ACN in one minute. All of the samples were filtered with a 0.45 μm micro-porous filter. The PDA wavelengths ranged from 210 to 500 nm, and the monitoring wavelength of the EG was 272 nm. The flow rate was 1 mL/min. The injection volume was 10 μL, and the column temperature was room temperature.

### 3.4. Bacterial Strain and Culture Conditions

*Streptococcus mutans* UA159 (700610; American Type Culture Collection, Manassas, VA, USA) was selected for this study because it preferably colonizes humans. Stocks of this strain were maintained in 10% skim milk (Difco; BD BioSciences, Franklin Lakes, NJ, USA) at −70 °C until use. Prior to the experiments, *S. mutans* was cultured in Bacto^™^ Todd Hewitt broth (THB; BD BioSciences) under anaerobic conditions (95% N_2_ and 5% CO_2_) at 37 °C for 18 h. The purity of the culture was checked on Mitis Salivarius agar (Difco; BD BioSciences, NJ, USA) and Columbia agar, with 7% sheep blood (E&O Laboratories, Bonnybridge, Scotland).

### 3.5. Microdilution Test for Determining the Minimum Inhibitory Concentration (MIC) and Minimum Bactericidal Concentration (MBC) 

The broth microdilution assay was performed using twofold serial dilution in BHI broth. The test was carried out in 96-well, flat-bottomed microtitration plates. The cell suspension was prepared in BHI broth with an optical density equivalent to 0.5 McFarland standard, and diluted 1:100 in BHI broth to obtain a final concentration of 10^6^ clone-forming units per milliliter (CFU/mL). Controls with broth only, and broth with bacteria but containing no antibacterial agents, were also included in each plate. One-hundred µL of antibacterial agent was put in the first microplate well and serially diluted in BHI broth. 100 µL, corresponding to 10^6^ CFU/mL, was added to all the wells. The plates were incubated at 37 °C for 18 h overnight. A twofold dilution of erythromycin was used as positive control. The MIC was defined as the lowest concentration able to inhibit the visible growth of bacteria in triplicate wells. After incubation, the MIC of ethyl gallate was determined to be the lowest concentration at which no growth was observable in the duplicate wells. After the MIC and MBC values were visually determined, 20 µL of *p*-iodonitrotetrazolium violet (8 mg/mL in ethanol) were added to each well. The plate was incubated for another 30 min and inspected visually for any change in color from yellow to pink, indicating a reduction of dye due to bacterial growth. The highest dilution (lowest concentration) that remained yellow corresponded to the MBC.

### 3.6. Biofilm Formation and Treatments

To evaluate the effectiveness of the treatments, *S. mutans* biofilm formation was induced on polystyrene and glass surfaces in separate experiments. Prior to each experiment, the optical density (OD) of the bacterial culture was adjusted to 0.2 at 630 nm in order to obtain the bacterial cell number of 1.6 × 10^8^ cells/mL, using a microplate-reader spectrophotometer. For the biofilm formation on polysterene surfaces, 24-well, flat-bottomed, polystyrene cell culture plates (Sarstedt, Nümbrecht, Germany) were filled with THB containing 1% sucrose, and then a solution of EG (Sigma-Aldrich, Merck KGaA, Darmstadt, Germany), prepared in sterile distilled water (Milli-Q water, Merck KGaA, Darmstadt, Germany), was added to the appropriate wells at final concentrations of 0.55 mg/mL (2.78mM), 0.58 mg/mL (2.93 mM), 0.61 mg/mL (3.08 mM), 0.64 mg/mL (3.23 mM), 0.67 mg/mL (3.38 mM), and 0.7 mg/mL (3.53 mM). *S. mutans* bacteria were then added to the wells at a final dilution of 1:100, and all of the plates were incubated anaerobically at 37 °C for 24 h. Quantification of the resulting biofilm (biomass) was performed using a colorimetric assay. The same experimental procedures were used for biofilm formation on glass surfaces, except that sterile glass slides of 1-mm thickness, cut from standard microscope slides (76 × 26 mm; Thermo Fisher Scientific, Inc., Waltham, MA, USA), were inserted vertically into the plate wells prior to the inoculation of the bacteria. Quantitative assessment of the resulting biofilm was performed via an optical profilometry assay. In these experiments, plate wells without bacterial cells were used as blank controls, and untreated bacteria served as experimental controls. 

### 3.7. Colorimetric Assay

After 24 h of incubation, THB was discarded from the plates, the wells were rinsed with distilled water to remove loosely bound bacterial cells, and then adherent bacteria were fixed with 95% ethanol. To quantify the biofilm biomass, the fixed and air-dried *S. mutans* biofilm in the plate wells was stained with 1 mL/well of 0.01% crystal violet solution (Merck KGaA, Darmstadt, Germany) for 15 min, and then the bound dye was extracted using 1 mL/well of 33% acetic acid solution (Merck KGaA) for 30 min. Next, 200 μL of extracted dye solution from each well was transferred to the appropriate wells in an optically clear, flat-bottomed, 96-well microplate. The OD of the samples was measured at a wavelength of 595 nm with a microplate-reader spectrophotometer. Background staining was corrected for by subtracting the amount of the staining in the blank wells.

### 3.8. Optical Profilometry Assay

Following 24 h of incubation, the glass slides with adherent *S. mutans* biofilm were removed from the plate wells, air-dried, and further analyzed using a non-contact optical imaging profilometer Sensofar PLµ 2300 system (Terrassa, Spain) and by applying a 50X confocal objective with a view field of 253 × 190 μm. Primarily, six regions of the glass slide were scanned in order to evaluate the surface roughness of each slide. A vertical scratch was made on the glass surface with a scalpel in the middle of every slide covered with biofilm, and then five regions of the glass slide were scanned to assess the biofilm thickness for each slide, halfway from the bottom to the top of the visible biofilm. The bottom of the scratch served as a reference point for accurate measurement of the biofilm thickness. All the images were captured in vertical scanning mode, and the data collected from the images were further processed using Gwyddion software (version 2.50, Department of Nanometrology, Czech Metrology Institute, Brno, Czech Republic; http://gwyddion.net) to calculate the parameters for surface roughness and biofilm thickness. A median filter (10 pixels or 3 μm) was selected to remove errors of form and waviness. The root mean square for roughness (*R*_q_), the most critical parameter, was calculated to quantitatively evaluate the slide surface roughness, which is an indication of the adherence of bacteria. The *R*_q_ parameter is an average of the measured height deviations taken within the evaluation length and measured from the mean line. It represents the standard deviation of the surface profile heights, and it is calculated according to the ISO 4287/1-1997 standard with the following formula:

(1) $$Rq={\left(1/N\sum_{j=1}^{N}rj^{2}\right)}^{**1/2}$$

where *N* is the number of points within a sampling length, and *r_j_* is the height value at point *j*. To measure the biofilm thickness, which indicated the maturity of the biofilm, the height of the artificially produced vertical scratch on each slide with adherent bacteria was used. Calculation of the biofilm thickness involved generating a height distribution graph curve based on the entire area of the scanned region containing a scratch, followed by Gaussian function fitting, as defined by

*f*(*x*) = *y*_0_ + *a* exp[−(*x* − *x*_0_)^2^/b^2^] (2)

where *y*_0_ is the peak height, *a* is the amplitude (height) distribution, *x*_0_ is the peak position, and *b* is the standard deviation. The background for the parameters of surface roughness and biofilm thickness was corrected for by subtracting the *R*_q_ and thickness values of a blank glass slide.

### 3.9. Biofilm Acidogenicity 

*S. mutans* biofilm formation and treatments were performed using the same procedures described above. After 24 h of incubation, the biofilm growth medium (THB) was collected from the wells of all the plates and transferred to 1.5-mL microcentrifuge tubes. The pH of the *S. mutans* biofilm growth medium collected in the tubes was measured with a microelectrode InLab^®^ Micro Pro ISM^®^ connected to a bench-top pH meter SevenCompact^™^ S210-Bio (Mettler-Toledo GmbH, Greifensee, Switzerland) at room temperature. The microelectrode was calibrated using standard pH buffers (pH 4.01 and 7.00) prior to and following each measurement.

### 3.10. Analysis of Gene Expression

Overnight cultures of *S. mutans* grown in LB broth were diluted into fresh LB medium of 1% sucrose to obtain a final concentration of 0.5 × 10^5^ clone-forming units per milliliter (CFU/mL) and were equally distributed into 50-mL tubes (30 mL/tube). Different concentrations of ethyl gallate were added to each tube to reach final concentrations of 1.56 mg/mL and 0.39 mg/mL. Cultures without ethyl gallate was used as a control. Cells were grown in three wells of 6-well plate at 5 mL per well (15 mL in total), and the plates were incubated at 37 °C for 24 h Planktonic cells were collected separately and stored at −4 °C for further analysis. The attached cells (biofilm) were scraped from the wells and stored at −4 °C for RNA isolation.

### 3.11. RNA Isolation

After harvesting the bacteria by centrifuging the cultures at 4800× *g* for 10 min, the bacterial pellet was suspended in 500 µL of ice-cold phosphate buffer solution (PBS). The suspension was then centrifuged at 500× *g* for 10 min at 4 °C. This washing step was repeated twice. The pellet was resuspended in 700 µL of the GENEzol^™^ TriRNA Bacteria Kit (Geneaid Biotech Ltd., Taiwan) and subjected to 3 cycles of deep freezing at −80 °C, followed by thawing at room temperature to allow the lysing of the cells, and was subsequently processed as described by the manufacturer. The quantity and purity of the total RNA samples were assessed by ultraviolet spectroscopy using a DS-11 Spectrophotometer (DeNovix Inc., Wilmington, North Carolina, USA). 

### 3.12. Relative RT-qPCR for the Estimation of Biofilm-Associated Gene Expression Following EG Exposure

One microgram of total RNA was reverse-transcribed using modified MMLV RTase, random hexamer primers included in the qPCRBIO cDNA Synthesis Kit (PCR Biosystems Ltd, London, England). The qPCR was performed using an *Exicycler*^™^ 96 PCR system designed for real-time qPCR (Bioneer, Korea) with qPCRBIO SyGreen Mix (PCR Biosystems Ltd, London, England). The total reaction volume was 20 µL, the cDNA template quantity was 100 ng, and the final primer concentration was 400 nM for both the forward and reverse primers, in accordance with the manufacturer’s instructions. The cycling conditions were as follows: 5 min of initial denaturation at 95 °C, 40 cycles consisting of 15 seconds at 95 °C and 60 seconds at 60 °C, and a final melting curve program. For the *gbpB* gene, forward primer 5′-ATGGCGGTTATGGACACGTT-3′ and reverse primer 5′-TTTGGCCACCTTGAACACCT-3′ (24). For the *gtfB* gene, forward primer

5′-AGCAATGCAGCCAATCTACAAAT-3′ and reverse primer 

5′-ACGAACTTTGCCGTTATTGTCA-3′ were used (5). For the *gtfC* gene, the forward primer

5′-GGTTTAACGTCAAAATTAGCTGTATTAGC-3′ and the reverse primer 

5′-CTCAACCAACCGCCACTGTT-3′ were used (5). For the *gtf*D gene, the forward primer

5′-ACAGCAGACAGCAGCCAAGA-3′ and the reverse primer

5′-ACTGGGTTTGCTGCGTTTG-3′ were used (5). For the *atp*D gene the forward primer 

5′-CCAGGCGGTTCATTCATCTGAC-3′ was used and the and reverse primer 

5′-GGCGGGATTTCGGTATTTACTG-3′ (11), and for the *atpF* gene, the forward primer

5′-CGGCTAAAAGAACACTAAG-3′ and the reverse primer 

5′-CGGTCGTCTAAAAGATAAG-3′ (11). All of these were used under the cycling conditions mentioned above. 

Amplifications using total RNA that was not reverse-transcribed were performed to check for genomic DNA contamination, and no-template controls were included. The comparative ∆∆CT method of Livak for qPCR data was applied as a standard procedure in the analysis of the relative gene expression data. The *C*_T_ values obtained from the experimental RNA samples were normalized to the reference gene 16S rRNA, and the difference in the ∆*C*_T_ values (∆∆*C*_T_) between the samples of interest and the control samples was calculated [28].

### 3.13. Statistical Analysis

The data obtained from colorimetric, profilometric, and pH measurements were analyzed using SPSS version 23.0 (IBM Corp., Armonk, NY, USA). The differences between the control (untreated) and treatment groups were evaluated by applying a one-way analysis of variance, followed by a post hoc least-significant difference test for multiple comparisons. The data are presented as the mean ± standard error. A *p* value of less than 0.05 was considered to indicate a statistically significant difference. In the gene expression experiments, three independent experiments with two technical tests were conducted for each treatment (n = 6). The statistical analysis was performed using a one-way analysis of variance, followed by the Tukey–Kramer test, at a significance level of 0.05. The figures display the mean and standard deviations. Statistical significance is denoted by an asterisk.

## 4. Conclusions

Natural sources in general, and plants in particular, are rich in antibacterial phytochemicals that are able to inhibit the growth of pathogenic bacteria, especially streptococci. Special attention has recently been paid to identifying natural products that can combat *S. mutans*, due to its association to oral carcinoma. Special focus was given in this study to the inhibition of biofilm production, since it is considered an especially virulent action of *S. mutans* on tooth surfaces. It was revealed that all of the EG concentrations tested significantly suppressed *S. mutans* biofilm build-up on polystyrene and glass surfaces, along with acidogenicity, in a dose-dependent manner, compared to the untreated condition (*p* < 0.05). The highest concentration of EG (3.53 mM) reduced biofilm formation on polystyrene and glass surfaces by 68% and more than 91%, respectively, and prevented a decrease in pH levels by 95%. Due to its capability to significantly inhibit biofilm formation and the acidogenicity associated with *S. mutans*, EG might be used as an antibiofilm or anticaries agent for oral formulations to reduce the prevalence of dental caries.

In this study, we investigated the effects of EG treatment on six important genes involved in biofilm production by *S. mutans*. The results show that biofilm-producing bacteria treated with EG exhibited significant changes in gene expression for three genes—*gtfC, gtfB*, and *gbpB*—with 98.6, 47.5, and 13.8 increases in fold change, respectively. However, for three other genes tested (*gtfD*, *atpD* and *atpF*), the EG treatments did not produce any significant expression change in comparison with the control. In order to determine the direct effects of EG on gene expression in biofilm-producing cells of *S. mutans,* more studies will be conducted in the future to follow the expression of a large number of genes known to be involved in biofilm production and maintenance, on the genetic level as well as on protein level. 

## Figures and Tables

**Table 1:** Antibacterial activity of ethyl gallate (stock solution 100 mg/mL dissolved in DMSO), erythromycin (positive control, stock solution 10 mg/mL dissolved in DMSO), and DMSO (solvent).

Compound	MIC, mg/mL	MBC, mg/mL
Ethyl gallate	1.56	6.25
Erythromycin	0.0048	0.0097
DMSO	25	25

**Table 2:** The pH levels of the *S. mutans* biofilm growth medium after 24 h of incubation in the presence of 1% sucrose and different concentrations of ethyl gallate (EG).

Experimental Group	pH
Blank	7.35 ± 0.01 *
Control	4.12 ± 0.01
EG ( 2.78 mM)	6.1 ± 0.17 *
EG ( 2.93 mM)	6.54 ± 0.09 *
EG ( 3.08 mM)	6.77 ± 0.04 *
EG ( 3.23 mM)	6.89 ± 0.02 *
EG ( 3.38 mM)	6.97 ± 0.01 *
EG ( 3.53 mM)	7.02 ± 0.01 *

Data are presented in terms of the mean ± standard error from three independent experiments (*n* = 3–9).*p < 0.05 when compared to the control group.
